# Quantification of the antimalarial drug pyronaridine in whole blood using LC–MS/MS — Increased sensitivity resulting from reduced non-specific binding

**DOI:** 10.1016/j.jpba.2017.08.023

**Published:** 2017-11-30

**Authors:** Daniel Blessborn, Karnrawee Kaewkhao, Lijiang Song, Nicholas J. White, Nicholas P.J. Day, Joel Tarning

**Affiliations:** a Mahidol-Oxford Tropical Medicine Research Unit, Faculty of Tropical Medicine, Mahidol University, Bangkok, 10400, Thailand; b Centre for Tropical Medicine and Global Health, Nuffield Department of Clinical Medicine, University of Oxford, Oxford, UK

**Keywords:** Pyronaridine, Pyramax, Malaria, Whole blood, Bioanalysis

## Abstract

Malaria is one of the most important parasitic diseases of man. The development of drug resistance in malaria parasites is an inevitable consequence of their widespread and often unregulated use. There is an urgent need for new and effective drugs. Pyronaridine is a known antimalarial drug that has received renewed interest as a partner drug in artemisinin-based combination therapy. To study its pharmacokinetic properties, particularly in field settings, it is necessary to develop and validate a robust, highly sensitive and accurate bioanalytical method for drug measurements in biological samples. We have developed a sensitive quantification method that covers a wide range of clinically relevant concentrations (1.5 ng/mL to 882 ng/mL) using a relatively low volume sample of 100 μL of whole blood. Total run time is 5 min and precision is within ±15% at all concentration levels. Pyronaridine was extracted on a weak cation exchange solid-phase column (SPE) and separated on a HALO RP amide fused-core column using a gradient mobile phase of acetonitrile–ammonium formate and acetonitrile-methanol. Detection was performed using electrospray ionization and tandem mass spectrometry (positive ion mode with selected reaction monitoring). The developed method is suitable for implementation in high-throughput routine drug analysis, and was used to quantify pyronaridine accurately for up to 42 days after a single oral dose in a drug-drug interaction study in healthy volunteers.

## Highlights

• Sensitive and accurate method suitable for high-throughput routine analysis of pyronaridine in whole blood.
• Validated according to US FDA bioanalytical validation guidelines.
• Described impact on freeze and thaw stability using different anticoagulants.
• Described adsorption and carry-over issues and how to overcome them.

## Graphical abstract

**Figure:**
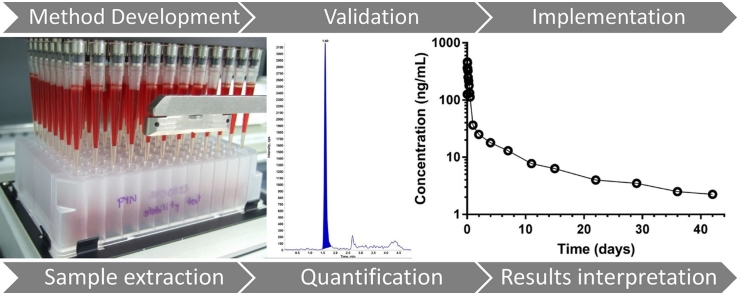

## 1 Introduction

Malaria is one of the most important infectious diseases in the world, resulting in an estimated 214 million cases and 438,000 deaths in 2015 [1]. Children under the age of five bear the main burden of malaria-attributed deaths. Artemisinin-based combination therapy (ACT) is the World Health Organization recommended first-line treatment against *P. falciparum* malaria [2]. The fast-acting artemisinin component kills the majority of parasites during the first days of treatment. As, this class of drug has a short biological half-life [3], [4], it is necessary to combine it with a long-acting partner drug to eliminate residual parasites and ensure cure. Pyronaridine is a synthetic antimalarial drug, developed in the 1970s, which has been used as a mono-therapy in China. It has a long biological half-life (13–17 days) and is therefore suitable to use in ACTs [5], [6], [7]. The fixed-dose combination of artesunate and pyronaridine is a newly developed and deployed ACT, which has shown excellent efficacy against both *P. falciparum* and *P. vivax* malaria [8].

Sensitive and accurate bioanalytical methods are crucial for high-quality pharmacokinetic studies. There are a few methods published for the quantification of pyronaridine in plasma [9], [10], [11], [12], and most of these require a sample volume of 200–250 μL of plasma and present a quantitation limit of 10 ng/mL or higher [11], [12]. Hodel et al. reported a LC–MS/MS method able to quantify pyronaridine at 1 ng/mL [10], but the run time was reported to be over 20 min making it unsuitable for high throughput routine analysis of clinical studies. An in vivo study in rabbits showed a high blood-to-plasma distribution ratio (4.9-17.8) for pyronaridine [13], suggesting that whole blood rather than plasma would be the preferred sample matrix for drug measurements [6], [14]. Several quantification methods have been reported for whole blood, but with a relatively poor sensitivity (i.e. 20–30 ng/mL) [13], [15], [16]. A recent LC–MS/MS method [17] used a sample volume of 300 μL, resulting in a lower limit of quantification (LLOQ) of 5.7 ng/mL. However, liquid-liquid extraction was employed which is laborious and difficult to automate. Another crucial drawback in the previously reported LC–MS/MS methods is that none of them used stable isotope-labeled internal standards, and could therefore be compromised by matrix effects.

The aim of this work was to develop a sensitive and accurate quantification method of pyronaridine in low volume whole blood samples that is suitable for implementation into high-throughput routine drug quantification in clinical trials.

## 2 Materials and methods

### 2.1 Materials

**Fig. 1:**
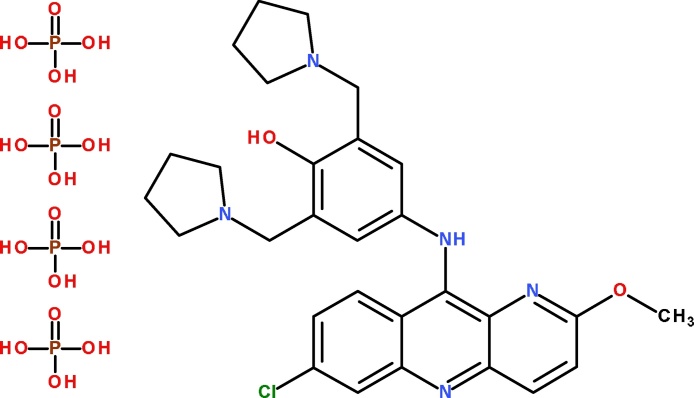
Chemical structure of pyronaridine tetraphosphate.

Pyronaridine tetraphosphate (Fig. 1) and its stable isotope labeled internal standard, pyronaridine-^13^C_2_D_4_ (SIL-PYN), was provided by the Worldwide Antimalarial Resistance Network Reference program [18]. LC–MS grade of water, acetonitrile and methanol were obtained from JT Baker (Phillipsburg, USA). All other chemicals and reagents were of analytical grade. Formic acid (98%), ammonium formate and ammonium carbonate were from Sigma–Aldrich (St. Louis, USA). Acetic acid was obtained from Merck (Darmstadt, Germany). Blank blood in fluoride-oxalate, EDTA, Na-heparin, Li-heparin, and fluoride-heparin was collected from healthy volunteers at the Faculty of Tropical Medicine, Mahidol University, Thailand.

### 2.2 Sample preparation

#### 2.2.1 Preparation of standards and working solution

Stock solutions (1 mg/mL) of pyronaridine and SIL-PYN were prepared in methanol-formic acid 1% (50:50 v/v) and stored in methanol-washed cryo vials at −80 °C. Working solutions were prepared in human plasma-water (50:50 v/v) and used for spiking of whole blood. All solutions were allowed to equilibrate to room temperature before use.

#### 2.2.2 Preparation of calibrators and quality control samples

Fresh fluoride-oxalate whole blood was used to prepare calibration standards at a concentration range of 1.47–882 ng/mL and LLOQ at 1.47 ng/mL, upper limit of quantification (ULOQ) at 882 ng/mL and over-curve samples (OC) at 1807 ng/mL. Quality control (QC) samples at 4.67, 57.2 and 452 ng/mL were prepared from a second stock solution. The final volume of working solution in whole blood was less than 5% in all samples.

#### 2.2.3 Extraction procedure

Sample preparation and SPE was performed on a Freedom Evo 200 platform (TECAN, Mannedorf, Switzerland). Pipette tips, 96-well plates and seal mats were all methanol-washed before use. Whole blood samples (100 μL) were manually aliquoted to the 96-well plate. Then, by liquid handler, 100 μL plasma-water 50:50 v/v was added in the first well (i.e. double blank) and 100 μL of internal standard (30 ng/mL) in plasma-water 50:50 v/v was added to all other wells. Four hundred microliters of ammonium carbonate (20 mM, pH 8.8) was added to all wells and then covered with a seal mat. The 96-well plate was mixed for 2 min at 1000 rpm and centrifuged for 2 min at 1100 × g. Then 500 μL of the centrifuged sample was transferred to a 96 well plate solid phase CBA extraction plate (Biotage, Uppsala, Sweden) and extracted according to the following procedure; (1) conditioning of the SPE cartridge with 1 mL methanol followed by 1 mL ammonium carbonate (20 mM, pH 8.8), (2) loading of the whole blood sample (500 μL), (3) washing with 1 mL ammonium carbonate (20 mM, pH 8.8) followed by 1.5 mL methanol-ammonium carbonate 20 mM (80:20 v/v) and finally 1 mL methanol-water (50:50 v/v), (4) elution with 0.9 mL methanol-formic acid (98:2 v/v). The eluates were evaporated under a gentle stream of nitrogen at 70 °C (Caliper TurboVap^®^ 96) until dryness and reconstituted in 400 μL mobile phase A (see below section 2.3.1). The extracted and reconstituted samples were mixed for 2 min at 1000 rpm, centrifuged for 2 min at 1100 × g and placed in the autosampler to equilibrate for at least 30 min before analysis.

### 2.3 Instrumentation and chromatographic conditions

#### 2.3.1 Chromatography

The LC system was an Agilent 1260 infinity system consisting of a binary LC pump, a vacuum degasser, a temperature-controlled micro-well plate autosampler set at 4 °C and a temperature-controlled column compartment set at 40 °C (Agilent technologies, CA, USA). Data acquisition and processing were performed using Analyst 1.5.2 (Applied Biosystems/MDS Sciex, CA, USA). The compounds were analyzed on a Halo amide fused-core column (50 mm × 2.1 mm; I.D. 2.7 μm) with a Halo amide pre-column (5 mm × 2.1 mm; I.D. 2.7 μm) at a flow rate of 400 μL/min (Advanced Materials Technology, Wilmington, DE, USA). The mobile phase consisted of solvent A: acetonitrile–ammonium formate (10 mM with 1% formic acid) (10:90 v/v) and solvent B: acetonitrile–methanol (25:75 v/v). The mobile phase gradient was A: 0–1.5 min, B: 1.7–2.9 min and A: 3.1–5.0 min (with 0.2 min gradient switch) and total run-time of 5 min. The sample injection volume was 2 μL.

#### 2.3.2 Mass spectrometry

An API 5000 triple quadrupole mass spectrometer (Applied Biosystems/MDS Sciex) with TurboV ionization source interface operating in the positive ion mode was used for the MS/MS analysis. Ion spray voltage was set to 5500 V, with a drying temperature at 650 °C. The curtain gas was 30 psi and the nebulizer (GS1) and auxiliary (GS2) gases 45 and 55 psi, respectively. Quantification was performed using selected reaction monitoring for the transitions m/z 518.10–447.15 and 524.25–453.15 (collision energy of 23 V) for pyronaridine and SIL-PYN, respectively.

### 2.4 Validation

The assay was validated according to the US Food and Drug Administration (FDA) guidelines on bioanalytical method validation [19]. Accuracy and precision of the method was determined by analysis of 5 replicate sets of 5 concentrations (1.47, 4.67, 57.2, 452 and 882 ng/mL) analyzed in 4 separate runs. The ability to dilute over-curve samples was investigated at 1807 ng/mL and 1:4 dilution with blank blood. Overall accuracy was calculated as the mean of the relative accuracies (i.e. relative error compared to the nominal concentration) at each QC level. Precision of the method (within-run, between-run and total-variability) was calculated using analysis of variance (ANOVA) and expressed as the relative standard deviation (%RSD).

#### 2.4.1 Linearity, selectivity and recovery

Linearity was assessed using the calibration standards from the four separate runs. The most suitable regression model was chosen based on the accuracy of the back-calculated concentrations of the calibration curves and QC samples [20].

Selectivity was evaluated by analysis of six blank samples from six different blood donors, and using different anticoagulants (fluoride-oxalate, EDTA, Na-heparin, Li-heparin and fluoride-heparin). Potentially interfering antimalarial drugs (i.e. chloroquine, amodiaquine, artesunate, dihydroartemisinin, primaquine and piperaquine) were evaluated using post-column infusion and also a conventional LC run.

Recovery was determined by comparing the peak area of QC samples ( × 5 at each level) with that of extracted blank blood samples post-spiked to contain the same nominal concentration as the QC sample, simulating a 100% extraction recovery.

#### 2.4.2 Matrix effect and carry-over

Post-column infusion was performed with injection of blank extracted blood samples from six different donors. Blood from a single donor was also collected with different anticoagulants to evaluate potential matrix effects from the different anticoagulants. To calculate the matrix effect, the MS response from extracted blank samples (post-spiked) was divided with the MS response of the analyte in neat solution, at the same nominal concentration. A calculated matrix effect below 0.85 or above 1.15 would imply that a matrix effect was present. Carry-over effects were also investigated by injecting 3 blank samples (mobile phase) directly after five injections of extracted samples at the ULOQ. If the signal was more than 20% of that of LLOQ it would be classified as carry-over effects.

#### 2.4.3 Stability

Stability of pyronaridine in whole blood was investigated during 3 freeze/thaw cycles where samples were frozen at −80 °C and then thawed at room temperature for 2–3 h during each 24 h freeze/thaw cycle. Short term stability at room temperature (22 °C) and at 4 °C was investigated at 4 h, 24 h and 48 h. Long-term stability at −80 °C for 1 year for both spiked samples and stock solutions were evaluated. Stability was also investigated during different steps in the extraction process e.g. buffer pre-treated blood (before SPE extraction) 4 h at 22 °C, extracted samples (in elution solution) at 4 °C for 24 h, as evaporated samples stored at 4 °C for up to 72 h as well as stability in LC autosampler at 4 °C for up to 72 h. A heat deactivating test was evaluated at 60 °C for 1 h to inactivate any HIV viruses in the blood samples [21], [22], [23].

### 2.5 Clinical pharmacokinetic study with incurred sample reanalysis

This assay was used to quantify pyronaridine in whole blood samples as part of an open-label cross-over study (NCT01552330) to evaluate the potential pharmacokinetic drug-drug interactions between pyronaridine-artesunate and primaquine in 17 healthy Thai volunteers [5]. The drug was given as a single oral dose of artesunate-pyronaridine (Pyramax^®^, Shin Poong Pharmaceuticals, Republic of Korea), with and without primaquine (Thai Government Pharmaceutical Organization [Thai GPO], Bangkok, Thailand). Blood samples were collected at different time intervals until day 42. A total of 680 samples were analyzed. A total of 73 samples were selected for incurred sample reanalysis.

## 3 Results and discussion

The range of the calibration curve 1.47–882 ng/mL was based on published clinical pharmacokinetic patient data [14], [17] with an extended range to cover between-patient differences. In the drug-drug interaction study in healthy volunteers [5], fluoride-oxalate was used as an anticoagulant for all drugs in the study and this anticoagulant was therefore selected for the validation of pyronaridine.

Pyronaridine adsorbs readily to different surface materials, particularly to glass but also to some extent to plastic surfaces. This is a major problem when diluting stock solutions with water at low concentrations, affecting working solutions and internal standards which could lead to seriously biased results if not taken into consideration [24], [25], [26]. The adsorption issue was eliminated in the developed method by using diluted plasma for both working solutions and internal standard dilutions.

### 3.1 Sample preparation

It is important that the spiked samples, which are used to quantify the unknown clinical samples, mimic the study samples as closely as possible. A shift in pH or too much organic solvent may precipitate some of the blood components. Plasma-water (50:50 v/v) was used to prepare the working solutions from a stock solution containing methanol-formic acid 1%. The amount of stock solution added was kept low (<5%) to avoid any precipitation of plasma in the dilution step. The generated plasma-water working solution would then have minimal effect on the spiked whole blood.

For sample extraction, protein precipitation is quick and easy but it also releases many other components from the blood sample, potentially contaminating the detector and causing the LC–MS/MS system to suffer from matrix effects. Liquid-liquid extraction has been used in many previous methods for pyronaridine [9], [11], [13], [16], [17] but it can be complicated and laborious when running large number of clinical study samples. SPE with a weak cation exchange extraction column is the selective method producing a clean extract; this extraction method is fast and suitable for high throughput analysis when operated in the 96 well plate format. The SPE washing consisted of three steps; (1) buffer only to wash out remaining blood components before (2) a high methanol wash step and finally (3) water-methanol to wash out remaining buffer before adding the elution solvent. This generated a very clean extract suitable for LC–MS/MS analysis, thus preventing down time of the instrument.

### 3.2 Instrumentation and chromatographic conditions

It was challenging to find a suitable LC-column for pyronaridine that produced a symmetrical peak shape. In the published literature, most LC-UV methods used ion-pairing reagents to improve the peak shape, but this is often not compatible with MS-detection [27]. Most C18 and CN columns that were screened showed various degrees of peak tailing, but a good peak shape was obtained from the amide fused-core HALO column without using any additives. The MS was operated in the positive ion mode, which generated several abundant fragment ions of pyronaridine (518.1 m/z); 447.2 m/z, 376.2, 378.2 and 341.2 m/z (listed in decreasing order of abundance).

### 3.3 Validation

The assay was validated according to the US FDA guidelines on bioanalytical method validation. Accuracy and precision of the method was determined by ANOVA and the method showed satisfactory performance (RSD below 15% at all concentration levels) when quantifying pyronaridine-spiked fluoride-oxalate whole blood samples (Table 1).

**Table 1:** Accuracy and precision when quantifying pyronaridine in whole blood.

Sample	Concentration (ng/mL)	Within-run precision (%)	Between–run precision (%)	Total-run precision (%)	Accuracy (%)
LLOQ	1.47	9.83	14.8	10.9	1.45
QC 1	4.67	7.23	8.42	7.44	−1.87
QC 2	57.2	4.56	9.27	5.57	−0.38
QC 3	452	3.93	2.56	3.74	3.36
ULOQ	882	3.26	4.38	3.46	−3.27
OC (1:4)	1807	3.64	4.53	3.80	−0.89

Results are presented from the analysis of 5 replicates/day over 4 days at each concentration level, using spiked fluoride-oxalate whole blood samples. Within-, between- and total-run precision were obtained from an ANOVA. Accuracy was calculated as the mean of the relative accuracies (i.e. relative error compared to the nominal concentration) at each QC level. Precision and accuracy must be within ±15% for all control levels except at LLOQ which should be within ±20%.

#### 3.3.1 Linearity, selectivity and recovery

Different calibration models were evaluated. The simplest model that described the calibration curve adequately and returned high accuracy for back-calculated QC samples was ordinary linear regression with 1/x weighting. The developed method showed good selectivity with no interfering peaks when analyzing blank blood from six different donors and also when using different anticoagulants (fluoride-oxalate, EDTA, Na-heparin, Li-heparin and fluoride-heparin). Furthermore, injection of high concentrations of commonly used antimalarial drugs (i.e. chloroquine, amodiaquine, artesunate, dihydroartemisinin, primaquine and piperaquine) did not interfere with the quantification of pyronaridine (data not shown). Recoveries (extracted sample/post-extraction spiked sample) were in the range of 65–75% for pyronaridine in fluoride-oxalate whole blood, similar to that previously reported [13], [15], [16], [17].

#### 3.3.2 Matrix effect and carry-over

Matrix effect investigation with post-column infusion did not show any increase or decrease of the pyronaridine signal when injecting extracted blank blood from 6 different donors or when using different anticoagulants. Matrix effects were also investigated by extracting blank blood that was post-spiked and comparing the response with spiked neat solution. The calculations showed a post-spiked/spiked neat solution ratio close to 1 for all samples, proving no ion suppression or enhancement from any of the 6 different blood donors or from the different anticoagulants.

**Fig. 2:**
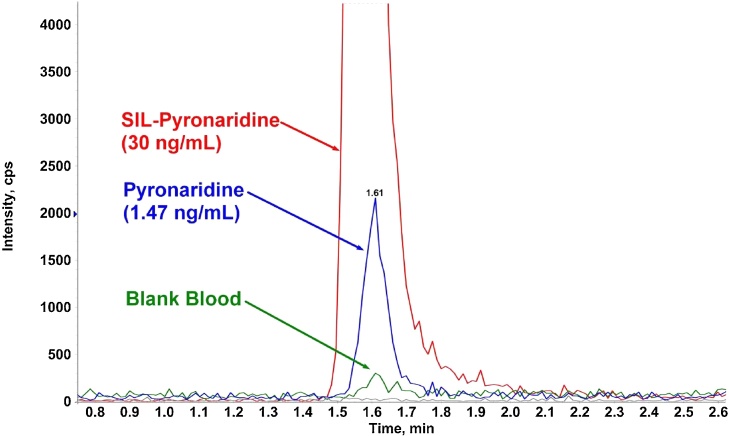
Overlay of blank human fluoride-oxalate blood and pyronaridine (1.47 ng/mL) with internal standard (SIL-PYN; 30 ng/mL) showing early signs of carry-over in the blank sample after about 80 sample injections.

Carry-over was noted after 150–250 samples (Fig. 2) and could not be eliminated with a more potent needle wash solution or longer wash time. The injector used a flow through design that should theoretically eliminate potential carry-over effects. However, the carry-over originated from contamination of the injection needle tip, and to solve this problem, a custom cleaning program was implemented between sample injections. The injection needle aspirated and dispensed a washing solution 4 times from each of the 4 wash-vials before aspirating the sample, followed by standard needle wash in a flush port for 15 s with acetonitrile-water-acetic acid (70:29:1 v/v/v) and injection of the sample onto the column. The 4 wash-vials contained the following solutions; Vial 1: acetonitrile:methanol 25:75; Vial 2: acetonitrile-formic acid (2% in water) 50:50 v/v; Vial 3: water only; Vial 4: mobile phase A. This custom wash successfully removed the carry-over problems but added almost 1 min to the run time of each sample.

#### 3.3.3 Stability

Freeze/thaw stability over three cycles showed good stability for spiked QC samples in fluoride-oxalate and EDTA. Fluoride-heparin barely passed the stability criteria, while Na-heparin and Li-heparin resulted in a 10–15% loss in each freeze-thaw cycle resulting in a total loss of 40–50% after three freeze-thaw cycles. There were no visual differences (precipitation or clogging) between cycles or samples that would explain such a significant drop. Thus, due to the poor freeze-thaw performance, Na-heparin and Li-heparin should not be used as anticoagulants for measurement of pyronaridine in blood samples.

Short term stability for up to 48 h in fluoride-oxalate blood at 22 °C and at 4 °C showed good stability without any degradation of the analyte. Long-term stability at −80 °C in the different anticoagulants showed good stability for up to 6 months. At 12 months the QC3 level was still within the 15% limit, but almost all measurements at the QC1 level were just outside the 15% limit (Table 2). However, after 50 months fluoride-oxalate samples are still within the acceptable range indicating some of the drop at 12 months could have been influenced by variation in the preparation of the fresh standards. Unfortunately no other anticoagulant samples were left for analysis at that time.

**Table 2:** Long-term stability of pyronaridine at −80 °C.

	Fluoride oxalate		EDTA		Li-heparin		Na-heparin		Fluoride-heparin	
	QC 1	QC 3	QC 1	QC 3	QC 1	QC 3	QC 1	QC 3	QC 1	QC 3
Day 0	100	100	100	100	100	100	100	100	100	100
2 months	106	101	107	103	100	103	99	104	101	105
6 months	89	95	96	100	98	99	89	99	92	93
12 months	83	97	89	97	84	87	80	86	81	92
50 months	89	90	N/A	N/A	N/A	N/A	N/A	N/A	N/A	N/A

The calibration curve was prepared in fluoride-oxalate. All other anticoagulant control samples were measured using this calibration curve, therefore all values has been normalized vs day 0 QC concentration measurements for each anticoagulant and are presented as percentages. Concentration level of QC 1 is 4.67 ng/mL and QC 3 is 452 ng/mL. N/A, Not available.

The stability of spiked fluoride-oxalate blood samples in the extraction process, i.e. buffer pre-treated blood waiting for extraction for up to 4 h at 22 °C, extracted samples in elution solution stored at 4 °C for up to 24 h, evaporated samples stored at 4 °C for up to 72 h, all showed acceptable stability profiles. The stability in the LC injector at 4 °C for up to 72 h and the heat deactivation at 60 °C for 1 h also showed good stability without any degradation of pyronaridine. The evaluated heat deactivation cannot deactivate all types of viruses but it will deactivate HIV viruses and hopefully reduce most other viruses making bioanalysis safer for laboratory workers [21], [22], [23]. Both pyronaridine and SIL-PYN stock solutions showed good stability when stored at −80 °C for up to 12 months.

### 3.4 Clinical applicability of the developed method

**Fig. 3:**
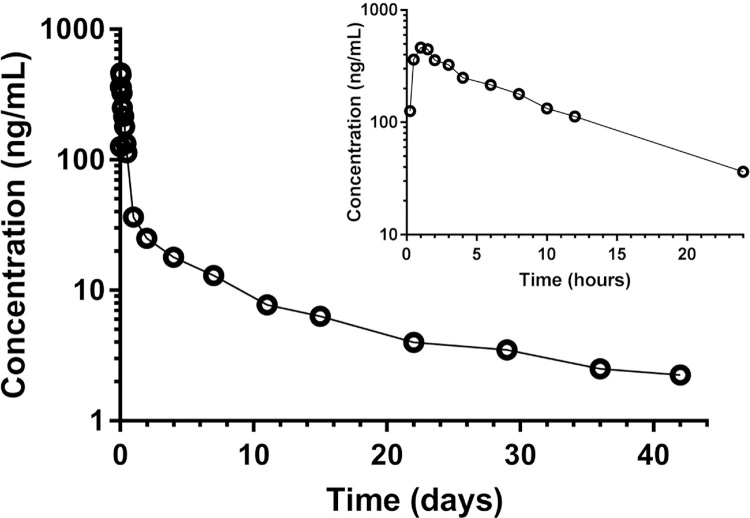
Observed concentration–time profile of pyronaridine after a single oral dose of 3 Pyramax^®^ tablets (180 mg pyronaridine tetra-phosphate and 60 mg artesunate per tablet) was given to a healthy adult Thai volunteer.

The developed and validated method was used to analyze a pharmacokinetic study in 17 healthy Thai volunteers [5]. Frequent blood samples were collected for up to 42 days after a single oral dose to evaluate the potential drug-drug interaction between artesunate-pyronaridine and primaquine. The developed method was implemented in a high-throughput setting, capable of analyzing up to 300 samples per day. The method showed excellent clinical applicability, covered the therapeutic range of observed concentrations, and was able to quantify clinical samples accurately with high sensitivity. Total precision (%CV) of quality control samples during analysis were 3.53%, 4.16% and 4.20% for QC1, QC2 and QC3. All day 42 samples (n = 31) were above the LLOQ concentration level. A concentration-time profile for a single healthy volunteer is shown in Fig. 3. A maximum concentration of 464 ng/mL was reached after 1 h and the drug was eliminated from the body with an elimination half-life of 17 days, resulting in a total exposure (AUC) of 11,100 (hr × ng/mL) in whole blood. Similar pharmacokinetic profiles could be seen for all volunteers.

To further test the reproducibility of the method, incurred sample reanalysis was performed on 73 out of 680 samples and the results were compared with the original data from the study. All samples were within the ±20% deviation limit and met the acceptance criteria for incurred sample reanalysis, proving a reproducible method free from any analytical issues.

## 4 Conclusions

The analytical method for pyronaridine in fluoride-oxalate whole blood passed the FDA validation criteria successfully and showed excellent accuracy and precision. The new method had much shorter run time (5 min) compared to previously published methods and used only 100 μL of whole blood. It provided a lower quantification limit of 1.5 ng/mL. However, there were a few findings that need to be highlighted. To avoid adsorption of pyronaridine or internal standard to surfaces, all working solutions used for dilution needed to contain a small volume of diluted plasma. Fluoride-oxalate and EDTA showed good freeze-thaw stability but Li-heparin and Na-heparin should be avoided due to the decreased recovery with subsequent freeze-thaw cycles. Carry-over issues in the HPLC-autosampler were overcome with the developed custom cleaning program that was implemented between sample injections.

Whole blood samples from a healthy volunteer study showed that the calibration curve covered the whole concentration range from the study subjects and that the method could quantify pyronaridine for up to 42 days after a single oral dose. The smaller sample volume of 100 μL compared to previously published methods will be a significant advantage in future field pharmacokinetic studies, particularly in the study of young children.
